# An Effective Hybrid Heterogeneous Catalyst to Desulfurize Diesel: Peroxotungstate@Metal–Organic Framework

**DOI:** 10.3390/molecules25235494

**Published:** 2020-11-24

**Authors:** Yan Gao, Fátima Mirante, Baltazar de Castro, Jianshe Zhao, Luís Cunha-Silva, Salete S. Balula

**Affiliations:** 1 REQUIMTE/LAQV & Department of Chemistry and Biochemistry, Faculty of Sciences, University of Porto, 4169-007 Porto, Portugal; gy746666512@stumail.nwu.edu.cn (Y.G.); fatimamirante@hotmail.com (F.M.); bcastro@fc.up.pt (B.d.C.); l.cunha.silva@fc.up.pt (L.C.-S.); 2 Key Laboratory of Synthetic and Natural Functional Molecule Chemistry of Ministry of Education, Shaanxi Key Laboratory of Physico-Inorganic Chemistry, College of Chemistry & Material Science, Northwest University, Xi’an 710127, Shanxi, China

**Keywords:** oxidation, desulfurization, metal–organic framework, peroxotungstate, MIL-101

## Abstract

A peroxotungstate composite comprising the chromium terephthalate metal–organic framework MIL-101(Cr) and the Venturello peroxotungstate [PO_4_{WO(O_2_)_2_}_4_]^3−^ (PW_4_) has been prepared by the impregnation method. The PW_4_@MIL-101(Cr) composite presents high catalytic efficiency for oxidative desulfurization of a multicomponent model diesel containing the most refractory sulfur compounds present in real fuels (2000 ppm of total S). The catalytic performance of this heterogeneous catalyst is similar to the corresponding homogeneous PW_4_ active center. Desulfurization efficiency of 99.7% was achieved after only 40 min at 70 °C using H_2_O_2_ as an oxidant and an ionic liquid as an extraction solvent ([BMIM]PF_6_, 2:1 model diesel/[BMIM]PF_6_). High recycling and reusing capacity was also found for PW_4_@MIL-101(Cr), maintaining its activity for consecutive oxidative desulfurization cycles. A comparison of the catalytic performance of this peroxotungstate composite with others previously reported tungstate@MIL-101(Cr) catalysts indicates that the presence of active oxygen atoms from the peroxo groups promotes a higher oxidative catalytic efficiency in a shorter reaction time.

## 1. Introduction

The significant increase in fuel consumption that has occurred in recent decades has brought convenience for people’s lives and for industrial production, but at the same time has caused environmental problems such as smog and acid rain [1,2]. To avoid these problems, a series of environmental protection regulations have been introduced including limitations in the content of sulfur in fuels to low-sulfur levels (10 ppm) [3,4]. Aromatic organosulfur compounds, as one of the main pollutant sources, widely exist in liquid fuel, and the sulfur oxides produced by fuel combusting are the main source of environmental pollution [5]. Hydrogenation, oxidation, extraction, adsorption, biodegradation and alkylation technologies have been developed to produce ultra-low-sulfur fuel and reduce environmental pollution [6,7,8,9,10]. Hydrodesulfurization (HDS) is the most widely used method; however, this needs to be operated under severe conditions (300–400 °C) and high hydrogen pressure (30–70 atm) to achieve high efficiency in removing refractory aromatic sulfur-containing compounds (e.g., dimethylbenzothiophene and derivates) from fuels [11,12,13]. In order to avoid the disadvantages of HDS, efforts have been made to find an effective alternative, and the oxidation desulfurization (ODS) process stands out as one promising method because of its advantages of mild reaction conditions, low cost, simple operation and high efficiency [14,15,16]. In ODS research, organosulfur compounds are oxidized and extracted by a polar solvent to obtain sulfur-free fuels. A desulfurization method combining extraction and oxidation has become potentially one of the deepest desulfurization technologies due to its higher efficiency and easier operation [15,17,18,19].

At present, most efforts are devoted to finding efficient catalysts and an ideal oxidant in order to obtain an effective and sustainable desulfurization technology. So far, a large number of studies have shown that polyoxometalates (POMs) as catalysts have high activity in oxidative desulfurization [20,21,22]. POMs with Keggin structures ([XM_12_O_40_]*^n^*^−^) are some of the most active for ODS processes. However, their high activity is in most cases is due to the transformation of the Keggin structures into peroxo-compounds, such as the Venturello structure ([PO_4_{WO(O_2_)_2_}_4_]^3−^, abbreviated as PW_4_), formed by the interaction with oxidants [23,24]. Therefore, the Venturello structure contains already active peroxo groups that can easily promote sulfur oxidation. In fact, the Venturello peroxotungstate, PW_4_, has been successfully used as a highly active catalyst in different oxidative reactions [15,24,25,26,27,28,29]. However, most of these studies use the Venturello compound as a homogeneous catalyst, which is difficult to recover and reuse [30]. However, the reusability of the catalyst is crucial to achieve cost-efficiency of the process. Therefore, it is of great significance to develop heterogeneous catalysts in practical production. The heterogenization of peroxotungstate can be achieved using various solid supports, such as the previous reported mesoporous silica materials (M41S, HMS-*n*, SBA-*n*) [15]. Nevertheless, metal–organic frameworks (MOFs) are also promising hosts for encapsulating polyoxometalates due to their advantages of high specific surface area, mesoporous cavity and stable structure [31,32,33]. MIL-101(Cr) is an excellent support material, which has high specific surface area, pore volume, and thermal (up to 300 °C) and chemical stability to water and organic solvents [34].

In the present work, the Venturello peroxotungstate (PW_4_) and a composite based on PW_4_ immobilized into MIL-101(Cr) support (PW_4_@MIL-101(Cr)) were successfully used as homogeneous and heterogeneous catalysts in oxidative desulfurization of model diesel. Eco-sustainable conditions (low temperature, low H_2_O_2_/S molar ratio and an environmentally friendly solvent (the ionic liquid [BMIM]PF_6_)) have been employed. The stability, the recycling and reusing capacity of the composite were investigated.

## 2. Results and Discussion

### 2.1. Characterization

The peroxotungstate (nBu_4_N)_3_{PO_4_[WO(O_2_)_2_]_4_}·6H_2_O (PW_4_) and MIL-101(Cr) were synthesized according to the reported literature with minor modifications [15,34,35]. A new catalyst (PW_4_@MIL-101) was prepared via a facile impregnation method incorporating the peroxotungstate into the MOF cavities, with a PW_4_ loading of 0.23 mmol/g (evaluated by ICP-OES). The crystalline phase, apparent morphology and thermal stability of the materials were analyzed by Fourier transform infrared spectroscopy (FT-IR), powder X-ray diffraction (PXRD), scanning electron microscopy (SEM) and thermogravimetric analysis (TGA).

**Figure 1:**
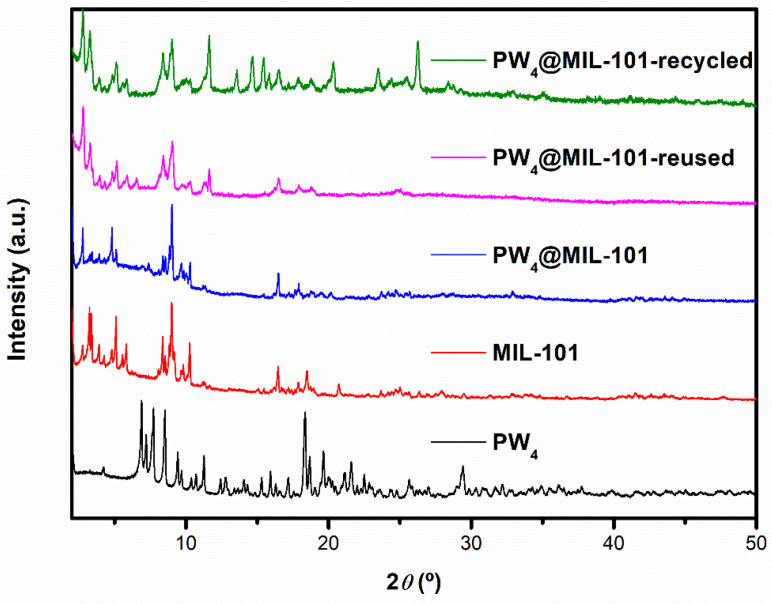
PXRD patterns of PW_4_ (black), MIL-101(Cr) (red) and PW_4_@MIL-101(Cr) (blue).

The phase purity of prepared MIL-101(Cr) was investigated by PXRD, with the diffraction pattern revealing good agreement with the pattern previously reported in the literature (Figure 1) [36]. The diffractogram of PW_4_@MIL-101(Cr) was similar to that of the parent material MIL-101(Cr), which confirmed that the framework structure of MIL-101(Cr) was practically undamaged during the process of embedding the PW_4_. In addition, the PXRD pattern of PW_4_@MIL-101(Cr) did not show any of the main diffraction peaks of PW_4_, which indicated that the PW_4_ was evenly dispersed in the cavity of MIL-101(Cr) and can be applied as a single active site to improve catalysis.

**Figure 2:**
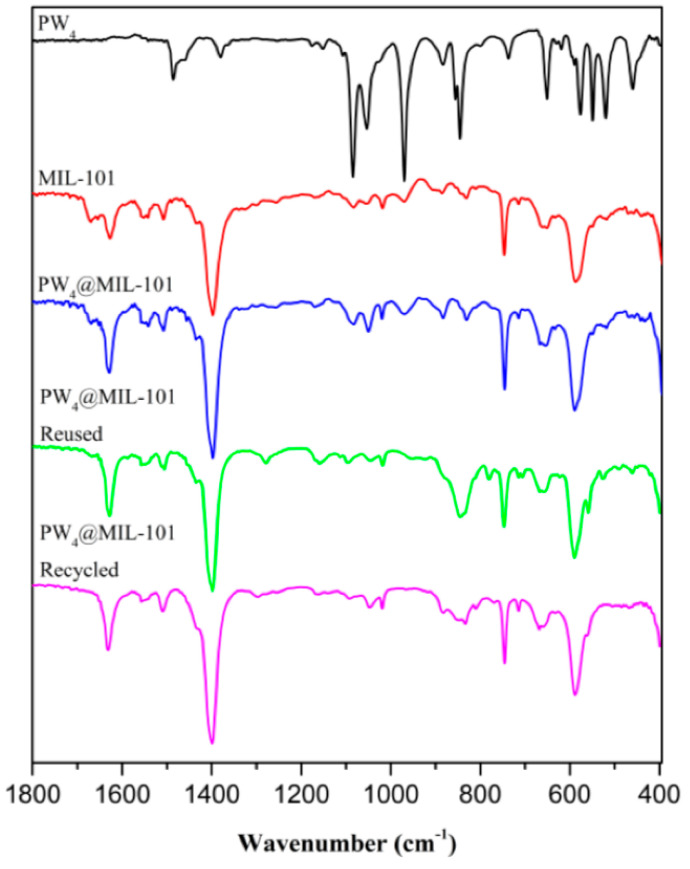
FT-IR spectra of PW_4_ (black), MIL-101(Cr) (red) and PW_4_@MIL-101(Cr) (blue).

The pristine and composite materials were further characterized by FT-IR (Figure 2). The spectrum of the composite PW_4_@MIL-101(Cr) reveals the same main characteristic peaks as MIL-101(Cr), namely at ca. 1632, 1399, 747 and 591 cm^−1^, corresponding to the stretching vibrations of C=C, COO^−^, C−H and Cr−O, respectively. The vital and strong bonds assigned to PW_4_, for example at 1086 cm^−1^ (asymmetric P−O_a_ stretch), 1054 cm^−1^ (symmetric P−O_a_ stretch), 970 cm^−1^ (W=O_d_ stretching mode), 846 cm^−1^ (O−O stretching vibration), 577 cm^−1^ (W−O_b_−W stretching vibration) and 551 cm^−1^ (W−O_c_−W stretching mode), were not clearly evident in the spectrum of PW_4_@MIL-101(Cr). Besides the low loading amount, this fact suggests a good dispersion of the PW_4_ into the cages of the MIL-101(Cr), and not a concentration on the surface of MIL-101(Cr).

**Figure 3:**
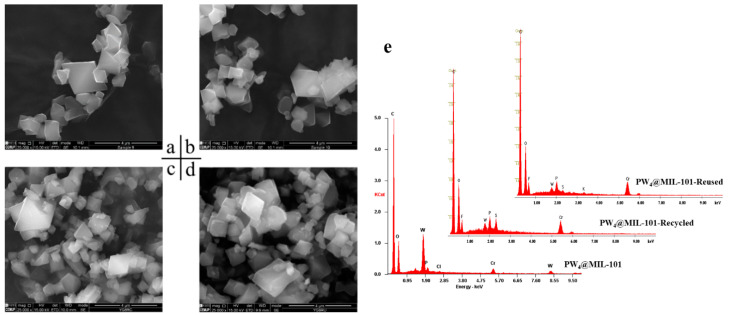
SEM images of pure MIL-101(Cr) (**a**), PW_4_@MIL-101(Cr) (**b**), PW_4_@MIL-101(Cr)-recycled (**c**) and PW_4_@MIL-101(Cr)-reused (**d**) and EDS spectrum of PW_4_@MIL-101(Cr), PW_4_@MIL-101(Cr)-recycled and PW_4_@MIL-101(Cr)-reused (**e**).

The SEM images of MIL-101(Cr) and PW_4_@MIL-101(Cr) revealed that the typical octahedral crystal structure with the size of 0.8 to 3.8 μm of MIL-101(Cr) was well preserved after encapsulating PW_4_ into the cavity of support (Figure 3), corroborating the maintenance of the crystalline structure verified by PXRD analysis. In addition, the W and P characteristic elements detected by EDX further unequivocally confirmed that the PW_4_ was incorporated into MIL-101(Cr).

**Figure 4:**
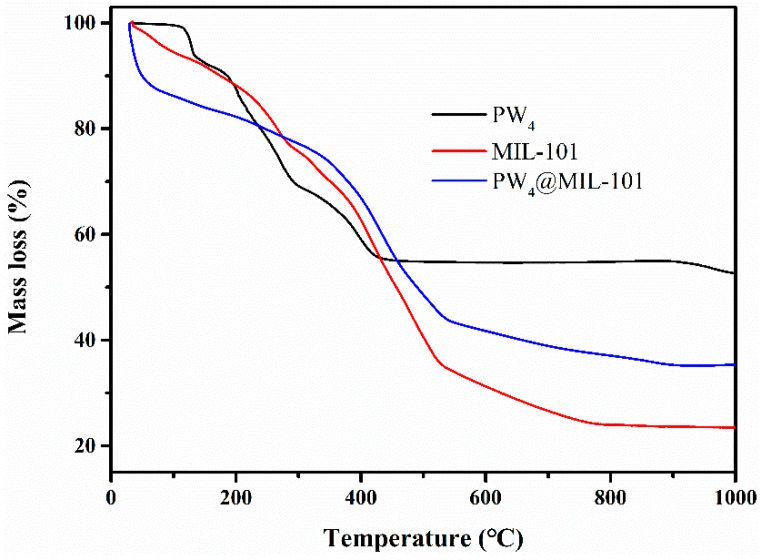
TGA curves for of PW_4_ (black), MIL-101(Cr) (red) and PW_4_@MIL-101(Cr) (blue).

The thermal stability of the materials was investigated by TGA (Figure 4). The TGA curve of PW_4_ revealed mass loss steps corresponding to water loss, decomposition of cation and anion, and dehydration–condensation reaction, with total weight loss of 45.1%, and above 400 °C, the POM decomposition to P_2_O_5_, WO_3_ and WO occurred [37]. In the case of MIL-101(Cr), the first mass loss, from 30 to 285 °C (loss of 22.9%), was due to the removal of the guest solvent (water, ethanol and/or DMF). The second step from 285 to 525 °C (loss of 41.3%) was assigned to the degradation of benzenedicarboxylate (BDC) linkers, and the last mass loss step (from 525 to 900 °C with mass loss of 10.85%) corresponded to additional degradation of the MOF. The TGA curve of PW_4_@MIL-101(Cr) was similar to that of the pristine MIL-101(Cr) except that the total weight loss (65.03%) was lower than that of MIL-101(Cr) (75.11%). According to total weight loss result, the loading amount of PW_4_ was calculated as 33.5%, which was consistent with the ICP-OES result.

### 2.2. Extraction and Catalytic Oxidative Desulfurization (ECODS)

**Scheme 1:**
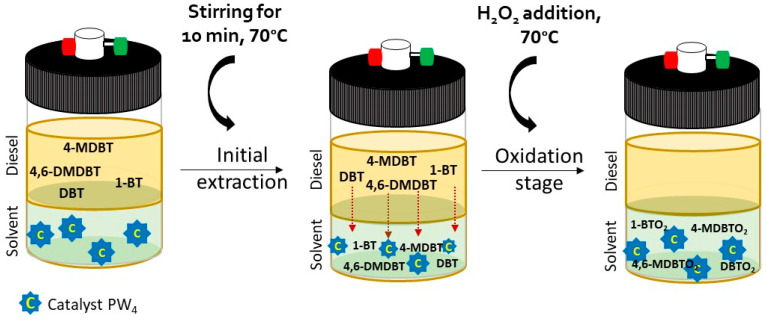
Illustration of the desulfurization conciliating extraction and oxidative catalytic processes using the catalyst PW_4_.

The pure PW_4_ and the composite PW_4_@MIL-101(Cr) were used as homogeneous and heterogeneous catalysts to desulfurize the model diesel containing four refractory aromatic sulfur compounds, namely, benzothiophene (BT), dibenzothiophene (DBT), 4-methyldibenzothiophene (4-MDBT) and 4,6-dimethyldibenzothiophene (4,6-DMDBT), with a total sulfur concentration of 2000 ppm. The extraction-oxidation desulfurization process was performed in a biphasic system formed by model diesel and extraction solvent (the ionic liquid IL, [BMIM]PF_6_) with a ratio of 2:1. The ECODS process mainly includes two steps: initial extraction under stirring for 10 min at 70 °C to achieve an extraction equilibrium of sulfur compounds that are transferred from the model diesel to the [BMIM]PF_6_, and the catalytic oxidation of the sulfur compounds present mainly in the extraction phase. By conciliating extraction and oxidation processes, a deep desulfurization can be achieved (Scheme 1). After the initial extraction, the oxidant H_2_O_2_ was injected into the system (0.26 mmol) to initiate the catalytic oxidation stage. The catalytic oxidation of sulfur compounds only occurs in the extractive phase due to the catalyst being dispersed in this layer, and no corresponding oxidative products were found in the model diesel phase due to the high solubility of the oxidized products in [BMIM]PF_6_. When the conversion of the oxidation of sulfur compounds increases, more non-oxidized sulfur compounds are transferred from the model diesel phase to the extraction phase. Therefore, the desulfurization system studied here combines a diffusional and a kinetic regime.

**Figure 5:**
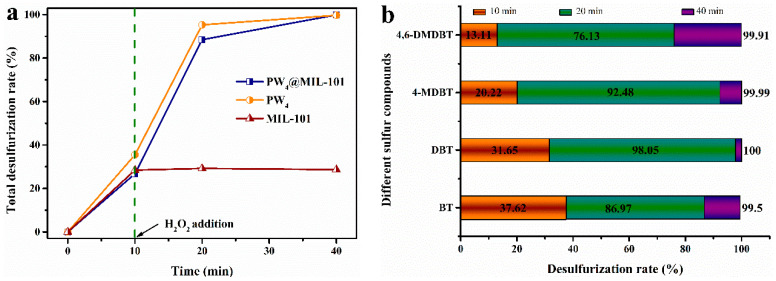
Desulfurization of the multicomponent model diesel (2000 ppm S) using PW_4_ (2.5 μmol) as a homogeneous catalyst, or PW_4_@MIL-101(Cr) as a heterogeneous catalyst (containing 2.5 μmol PW_4_), H_2_O_2_ oxidant (0.26 mmol) and [BMIM]PF_6_ as an extraction solvent (2:1 model diesel/[BMIM]PF_6_) at 70 °C (**a**) and desulfurization efficiency of each sulfur compound in the presence of PW_4_@MIL-101(Cr) catalyst (**b**).

The desulfurization results catalyzed by homogeneous PW_4_ and corresponding heterogeneous composite PW_4_@MIL-101(Cr) show practically complete desulfurization of the model diesel, obtaining 99.71% and 99.83% desulfurization after only 40 min, respectively (Figure 5a). It is clearly that the homogeneous PW_4_ catalyst is slightly more efficient than heterogeneous PW_4_@MIL-101(Cr) during the first 20 min of the desulfurization process. After 40 min, the desulfurization efficiency catalyzed by the homogeneous and heterogeneous catalysts is practically the same. This fact must be due to the large cavities of the MIL-101(Cr) that can accommodate the PW_4_ in its free form, which will enable PW_4_ to interact with the substrate and oxidants as when it is in its homogeneous form. In addition, the desulfurization test catalyzed by the support MIL-101(Cr) did not present any catalytic activity. Moreover, the complete desulfurization of model diesel was not achieved using the stoichiometric amount of oxidant H_2_O_2_/S = 2 (94% after 40 min of reaction and 96% after 2 h). Therefore, an excess of oxidant was needed to achieve complete desulfurization (H_2_O_2_/S = 4). This is probably due to the occurrence of a slight non-efficient decomposition of the oxidant, maybe caused by the temperature effect.

Figure 5b shows the desulfurization of the individual sulfur compounds during the different steps of the desulfurization process, i.e., initial extraction (after 10 min) and oxidative catalytic desulfurization (after 20 min and 40 min) using the composite PW_4_@MIL-101(Cr). The initial extraction efficiency of the studied organic sulfurs decreases in the order BT > DBT > 4-MDBT > 4,6-DMDBT, which is related to their solubility in the IL extraction phase. The sequence of the overall desulfurization efficiency of those refractory sulfur compounds is DBT > 4-MDBT > 4,6-DMDBT > BT, which is consistent with previously reported works based on polyoxometalate catalysts and H_2_O_2_ as an oxidant [15,18,30]. It is acceptable that oxidation of sulfur is affected by electron density and steric hindrance of sulfur atoms. That is, low atomic density and large steric hindrance have a negative effect on sulfur oxidation. In this work, DBT and its methyl-derivatives (4-MDBT and 4,6-DMDBT) have similar sulfur atom electron density (5.758, 5.759 and 5.760, respectively); however, the presence of methyl substituent leads to an increase in steric hindrance and makes oxidation of sulfur difficult. For BT, it has no large steric hindrance, but the low electron density of the sulfur atom (5.739) results in the lowest activity. Similar results for individual sulfur compounds desulfurization was achieved in the presence of homogeneous and heterogeneous catalysts. Therefore, the application of a heterogeneous catalyst that has identical high catalytic activity as its analogous homogeneous type is considered to be of crucial importance because of its advantages of easy recovery and reuse.

### 2.3. Reusability and Recyclability of PW_4_@MIL-101(Cr) Catalyst

**Figure 6:**
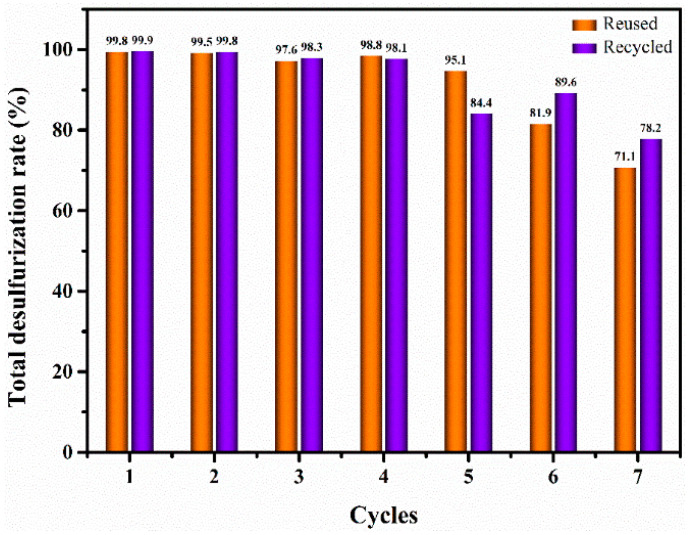
Results of desulfurization obtained for consecutive reusing and recycling ECODS cycles after 40 min of reaction, catalyzed by PW_4_@MIL-101(Cr) (containing 2.5 μmol PW_4_), H_2_O_2_ oxidant (0.26 mmol) and [BMIM]PF_6_ as the extraction solvent (2:1 model diesel/[BMIM]PF_6_) at 70 °C.

The recyclability and reusability of the composite PW_4_@MIL-101(Cr) were investigated for various consecutive ECODS cycles (Figure 6). For the study of recyclability, the solid catalyst was recovered by centrifugation at the end of each ECODS cycle, washed several times with MeCN, dried at room temperature and used in the next ECODS cycle by adding extraction solvent ([BMIM]PF_6_), model diesel and the oxidant (after 10 min). In the reusing study, the desulfurized model diesel was separated from the extraction solvent ([BMIM]PF_6_) and the solid catalyst at the end of each ECODS cycle. A new cycle was then performed by adding a novel portion of model diesel to treat and the oxidant (after 10 min).

The results displayed in Figure 6 demonstrate that the recycling and reusing behavior of the catalyst is similar during the seven ECODS cycles, and only a slight loss of desulfurization efficiency is observed after the fourth cycle.

Therefore, a deep desulfurization could be achieved using PW_4_@MIL-101(Cr) catalyst with desulfurization efficiency higher than 95% for five consecutive ECODS cycles, even maintaining the same portion of [BMIM]PF_6_ extraction solvent. The decrease in desulfurization efficiency observed after the fifth cycle must be due to some deactivation of the solid catalyst. This must be related to the adsorption of oxidized sulfur compounds on the surface of the solid catalyst and/or to a slight leaching of PW_4_ active center.

### 2.4. Stability of the Catalyst

The heterogeneous catalyst, after being reused and recycled for seven consecutive cycles (PW_4_@MIL-101(Cr)-reused and PW_4_@MIL-101(Cr)-recycled), was characterized through several techniques to analyze its stability. The FT-IR spectra of the reused and recycled composite are similar to the corresponding spectra of fresh catalyst, showing the characteristic peaks of MIL-101(Cr) (Figure 2). From the comparison of the SEM micrographs of the PW_4_@MIL-101(Cr) composite with the recycled and reused solid catalyst (Figure 3a–c), it is possible to observe the maintenance of the particles’ morphology. The EDX analysis of the reused and recycled samples presents the P and W elements from the active center PW_4_ and the Cr from the support material. Furthermore, the S element is well identified in both reused and recycle composites, which indicates that PW_4_@MIL-101(Cr) presents some adsorptive capacity, probably for the sulfur oxidized products. From the powder XRD pattern of the reused and recycle samples (Figure 1), it is possible to observe the characteristic peaks (positions and intensities) of the pristine MIL-101(Cr). The pattern of the reused sample (PW_4_@MIL-101(Cr)-reused) is similar to the support and the pre-used PW_4_@MIL-101(Cr) sample. However, some extra peaks are clearly observed in the recycled sample (PW_4_@MIL-101(Cr)-recycled), mainly one more intense peak around 13° and less intense peaks in the region between 14° and 20° and around 26°. These extra peaks can be attributed to the presence of sulfone compounds adsorbed on the PW_4_@MIL-101(Cr)-recycled sample [38], corroborating the results of the previous EDX analysis. The ICP-OES analysis of the solid catalyst after ECODS cycles presents that the W/Cr ratios of reused and recycled materials were 0.89 (wt%) and 0.77 (wt%), which are lower than the correspondent ones before catalysis (1.47 wt%). These results indicate that some leaching of active center of PW_4_ may have occurred during the seven consecutive cycles, which caused the observed decrease in catalytic efficiency, mainly observed after the fifth cycle.

### 2.5. Comparison of Catalytic Efficiency

The catalyst efficiency of PW_4_@MIL-101(Cr) for oxidative desulfurization was compared with previously reported heterogeneous catalysts based on MIL-101(Cr) composites. In fact, various other polyoxometalates were previously incorporated into the porous MIL-101(Cr) structure, and these have been used as heterogeneous catalysts in ECODS systems. Most of these polyoxometalates are also derivated from the Keggin structure, like the Venturello PW_4_ compound: trivacant-phosphotungstate (PW_9_@ MIL-101(Cr)) [39], europium-sandwich type (Eu(PW_9_)_2_@MIL-101(Cr)) [18], terbium-sandwich type (Tb(PW_9_)_2_@ MIL-101(Cr)) [40], Keggin-type phosphotungstate (PW_12_@ MIL-101(Cr)) [41], phosphomolybdate (PMo_12_@MIL-101(Cr)) [42] and zinc-substituted phosphotungstate (PW_11_Zn@MIL-101(Cr)) [43]. Using these catalysts, complete desulfurization was achieved under similar experimental conditions, for a reaction time higher than 2 h using model diesel with similar sulfur content or even with less sulfur components and a total sulfur concentration not higher than 2000 ppm. Therefore, the application of a heterogeneous catalyst with an active center derived from POMs containing peroxo groups, i.e., with available active oxygen atoms to proceed with sulfur oxidation, without the need of previous formation of active intermediates or catalyst activation, is a better strategy to achieve high efficiency in a shorter reaction time.

## 3. Experimental Section

### 3.1. Materials and Methods

All the reagents used in peroxotungstate and metal–organic framework (MOF, MIL-101(Cr)) syntheses, namely, phosphotungstic acid hydrate (H_3_PW_12_O_40_·14H_2_O, 99.9%, Sigma-Aldrich, Darmstadt, Germany), tetrabutylammonium chloride (Bu_4_NCl, 98%, Sigma-Aldrich, Darmstadt, Germany) and hydrogen peroxide (H_2_O_2_, 30% *w/w* aq. Sigma-Aldrich, Darmstadt, Germany); and chromium(III) nitrate nonahydrate (Cr(NO)_3_·9H_2_O, 99%, Aldrich, Bethesda, MD, 20894 USA), benzene-1,4-dicarboxylic acid (C_8_H_6_O_4_, 98%, Aldrich, Darmstadt, Germany) and hydrofluoric acid (HF, 40–45%, Aldrich, Darmstadt, Germany), were used as received. The reagents for ODS studies, including 1-butyl-3-methylimidazolium hexafluorophosphate ([BMIM]PF_6_, Aldrich, Darmstadt, Germany) as an extraction agent and all reagents for the model diesel such as 1-benzothiophene (1-BT, Fluka, Darmstadt, Germany), dibenzothiophene (DBT, Aldrich, Darmstadt, Germany), 4-methyldibenzothiophene (4-MDBT, Aldrich, Darmstadt, Germany), 4,6-dimethyldibenzothiophene (4,6-DMDBT, Alfa Aesar, Kandel, Germany), decane (98%, Sigma-Aldrich, Darmstadt, Germany) and tetradecane (99%, Sigma-Aldrich, Darmstadt, Germany), were bought from chemical suppliers and used without further purification.

The materials were fully tested and characterized by different chemical analyses. Determination of the content of metal elements in the composite was carried out by ICP-OES on a Perkin-Elmer Optima 4300 DV instrument (University of Santiago de Compostela, Santiago de Compostela, Spain) to calculate the loading amount of PW_4_. Powder X-ray diffraction (XRD) data of all materials were collected at room temperature on a Philips X’Pert MPD diffractometer (Cu-Kα X-radiation, λ = 1.54060 Å, Faculty of Science, Porto, Portugal), equipped with an X’Celerator detector, a curved graphite monochromator and a plate sample holder in a Bragg–Brentano para-focusing optics configuration at 45 kV and 40 mA in the range of 2–50° by the step-counting method with the scanning speed of 0.1°/min. Infrared absorption spectra (FT-IR) were applied to detect functional groups in the 400–4000 cm^−1^ region using a Jasco 460 Plus spectrometer (Faculty of Science, Porto, Portugal) with 64 scans. The morphology of fresh and reused catalyst was observed on JEOL JSM 6301F (CEMUP, University of Porto, Porto, Portugal) at 15 kV with the magnification of 25,000×. More information about the elements in the catalysts was obtained by an energy-dispersive X-ray spectrometer (EDX, CEMUP, University of Porto, Porto, Portugal). The stability of the materials and the loading amount of the active component were further analyzed by thermogravimetric analysis under nitrogen atmosphere from room temperature to 1000 °C, with temperature rise rate of 10 °C·min^−1^. Catalytic reactions were periodically monitored by GC-FID analysis carried out in a Bruker 430-GC-FID chromatograph (Bruker, Faculty of Science, University of Porto, Porto, Portugal). Hydrogen was used as a carrier gas (55 cm·s^−1^), and fused silica Supelco capillary columns SPB-5 (30 m × 0.25 mm i.d.; 25 μm film thickness) were used.

### 3.2. Preparation of Catalysts

#### 3.2.1. Synthesis of Peroxotungstate

The preparation procedure to obtain peroxotungstate (nBu_4_N)_3_{PO_4_[WO(O_2_)_2_]_4_}·6H_2_O, marked as (PW_4_), was adapted from published procedures [15,44]. An aqueous solution of H_3_PW_12_O_40_·14H_2_O (1.44 g, 0.50 mmol was dissolved in 10 mL deionized water) was added to 30% aq. H_2_O_2_ (10 mL, 98 mmol), and the colorless solution turned to pale yellow and then became colorless again. After stirring for 60 min, 3 mL aqueous solution of tetrabutylammonium chloride nBu_4_NCl (0.45 g, 1.60 mmol) was introduced to the mixture above by dripping slowly and formed a white precipitate. Afterwards, the target material was obtained by filtration, washed with deionized water three times, dried and stored in a desiccator. The successful preparation of PW_4_ was confirmed by FT-IR, ^31^P NMR and elemental analysis. Yield: 0.9640 g (85.3%). Anal. Calcd. (%) for (nBu_4_N)_3_{PO_4_[WO(O_2_)_2_]_4_} 6H_2_O (1931,76): C, 30.01; H, 6.09; N, 2.22. Selected FT-IR (cm^−1^): 1483(w), 1381(w), 1086(s), 1053(m), 971(s), 882(w), 847(m), 738(w), 653(m), 578(m), 551(m), 522(m) and 459(m). ^31^P RMN 4.57 ppm (161.9 MHz, CD_3_CN, 25 °C).

#### 3.2.2. Preparation of Porous MIL-101(Cr)

The porous material MIL-101(Cr) was synthesized as described in the literature by the solvothermal method [34,35]. The reagents Cr(NO_3_)_3_ 9H_2_O (0.40 g, 1.00 mmol), terephthalic acid (0.166 g, 1.00 mmol) and hydrofluoric acid (100 μL) were added into H_2_O (10 mL) and stirred for 10 min at room temperature. The mixture was transferred to a teflon reactor and heated at 220 °C for 9 h using an electric oven. After the reaction had finished, the dark green product was isolated by filtration, washed by DMF and MeOH, and dried. FT-IR (cm^−1^): 1667(w), 1628(w), 1545(w), 1507(w), 1399(s), 1084(w), 1018(w), 972(w), 884(w), 835(w), 745(m), 658(w) and 583(m).

#### 3.2.3. Preparation of PW_4_@MIL-101(Cr) Composite

The composite was prepared by encapsulation of PW_4_ into MIL-101(Cr), which has a larger window size, by one of the most commonly applied methods described in the literature to impregnate POMs in MOFs [32]. A solution of PW_4_ (0.27 g, 0.14 mmol) in acetonitrile (10 mL) was added to the porous MOF-101(Cr) (0.44 g), previously dried by vacuum for 12 h at 60 °C. This mixture was stirred for 72 h at room temperature. The solid material PW_4_@MIL-101(Cr) was obtained by filtration, washed with acetonitrile and ethanol three times and dried. The loading amount of PW_4_ calculated by ICP-MS analysis was 0.23 mmol·g^−1^ (W/Cr ratio of 1.47 wt%). FT-IR (cm^−1^): 1628(m), 1541(w), 1508(w), 1397(s), 1050(w), 1018(w), 970(w), 883(w), 831(w), 746(m), 715(w), 655(w), 651(w) and 589(m).

### 3.3. Desulfurization of a Multicomponent Model Diesel

The simulated diesel with a total sulfur concentration of 2000 ppm was prepared with equal amounts of four refractory sulfur compounds present in real diesel: 1-benzothiophene (1-BT), dibenzothiophene (DBT), 4-methyldibenzothiophene (4-MDBT) and 4,6-dimethyldibenzothiophene (4,6-DMDBT) dissolved in decane. All the experiments were carried out under air (atmospheric pressure) in a closed borosilicate 5 mL reaction vessel, equipped with a magnetic stirrer and immersed in a thermostatically controlled liquid paraffin bath at 70 °C. The catalytic oxidative step was performed in the presence of an immiscible polar solvent, the ionic liquid 1-butyl-3-methylimidazolium hexafluorophosphate ([BMIM]PF_6_).

The oxidation of the sulfur compounds only occurred in the presence of a catalyst and an oxidant, where H_2_O_2_ (aq. 30%) was used as the oxidant, the Venturello compound PW_4_ as the homogeneous catalyst, and PW_4_@MIL-101(Cr) as the heterogeneous catalyst (2.5 μmol of active center PW_4_ was used). In a typical experiment, 0.75 mL of model diesel and the extraction solvent (0.38 mL) were added to the catalyst. An initial extraction of sulfur compounds from the model diesel to the extraction solvent phase occurred by only stirring both immiscible phases for 10 min at 70 °C. The oxidative catalytic step of the process was then initiated by the addition of H_2_O_2_ oxidant (0.26 mmol). The sulfur content in the model diesel phase was periodically quantified by GC analysis using tetradecane as standard. The recycle capacity of the heterogeneous catalyst (PW_4_@MIL-101(Cr)) was investigated using the same solid in seven consecutive desulfurization cycles. At the end of each desulfurization cycle, the catalyst was recovered, washed with acetonitrile 3 times and dried at room temperature. New portions of model diesel, oxidant and extraction solvent were added to the used catalyst to start a new desulfurization cycle. Furthermore, a reusing process was also performed, where the extraction phase containing either catalyst was reused without any further treatment, after removing the treated model diesel. A new reusing cycle is performed by only adding a new portion of sulfurized model diesel and H_2_O_2_ oxidant. All reused and recycled cycles were performed under the same initial experimental conditions. Experiments were repeated at least three times, and the estimated error was of 3% after the 40 min of total desulfurization (extraction and oxidation) and 5% after the initial extraction step.

## 4. Conclusions

A Venturello composite PW_4_@MIL-101(Cr) was successfully prepared by the impregnation of the peroxotungstate [PO_4_{WO(O_2_)_2_}_4_]^3−^ (PW_4_) into a porous MIL-101(Cr) support. This heterogeneous catalyst presents high efficiency in oxidative desulfurization of a multicomponent model diesel containing the maximum refractory sulfur compounds (2000 ppm of total S). The desulfurization process developed here conciliated extraction and catalytic oxidative desulfurization (ECODS). Similar catalytic behavior was found for the heterogeneous composite PW_4_@MIL-101(Cr) and the homogeneous Venturello, PW_4_. Desulfurization efficiency of 99.7% and 99.8% was achieved using heterogeneous and homogeneous catalysts, respectively, after 40 min at 70 °C, with H_2_O_2_ oxidant (0.26 mmol) and [BMIM]PF_6_ as the extraction solvent (2:1 model diesel/[BMIM]PF_6_). Furthermore, the PW_4_@MIL-101(Cr) composite presents high catalytic efficiency for at least five recycling ECODS cycles or even for five reusing ECODS cycles, maintaining some portion of extraction solvent during consecutive cycles and without the need for catalyst recovery and cleaning processes between cycles.
